# Protein kinase A: a quirky prototype

**DOI:** 10.1111/febs.70176

**Published:** 2025-07-02

**Authors:** Matthew G. Gold

**Affiliations:** ^1^ Department of Neuroscience, Physiology & Pharmacology University College London UK

**Keywords:** cAMP, dimer, neurodevelopmental disorder, protein kinase A

## Abstract

Protein kinase A (PKA) has served as a prototype for establishing kinase fundamentals, including sequence, structure and catalytic mechanism. However, PKA is quirky in some respects. Its regulatory elements are expressed separately, including type I (RI) regulatory subunits that contain unusual disulphide‐linked dimerization and docking domains. Benjamin‐Zukerman and colleagues report on the RIβ mutation L50R that disrupts this domain to cause neuronal loss and parkinsonism driven by a PKA mutation. They show that PKA catalytic subunits are released more easily from RIβ subunits containing the L50R mutation, adding depth to our understanding of this neurodevelopmental disorder.

## Graphical abstract

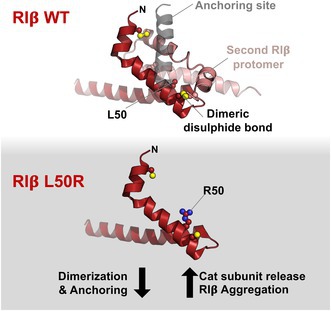

**Abbreviations**
AKAP: A‐kinase anchoring protein
C: catalytic subunit
cAMP: cyclic 3′‐5′ adenosine monophosphate
D/D: dimerization and docking
NLPD‐PKA: neuronal loss and parkinsonism driven by a PKA mutation
PKA: protein kinase A
RI: type I regulatory subunit
WT: wild‐type

## Introduction

**Fig. 1:**
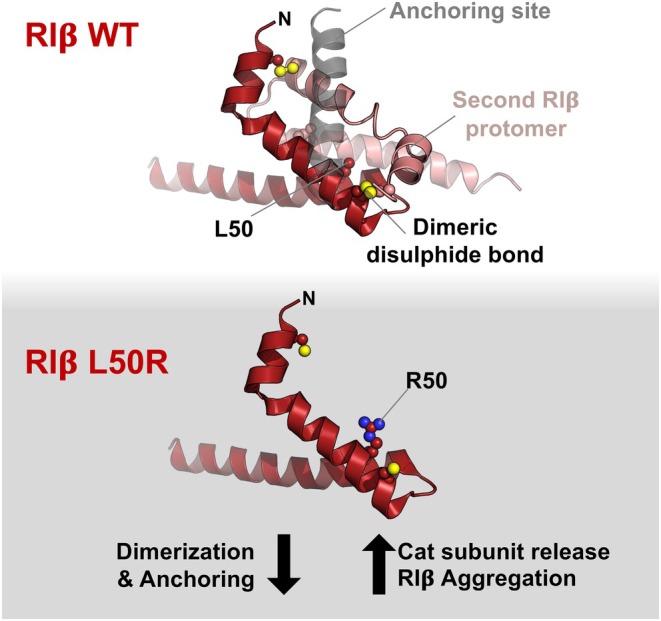
L50R substitution affects multiple aspects of RIβ function. L50R substitution affects multiple aspects of RIβ function. The docking and dimerization (D/D) domain of RIβ is shown for wild‐type (WT) (upper panel) and L50R (lower panel) variants. The RIβ D/D structure corresponds to PDB 4F9K—the position of the anchoring helix (grey) was determined by alignment to PDB 5HVZ. The L50R mutation undermines multiple aspects of normal RIβ function, leading to neuronal loss and parkinsonism driven by a PKA mutation (NLPD‐PKA) disease.

Cyclic 3′‐5′ adenosine monophosphate‐dependent protein kinase, also known as protein kinase A (PKA), has served as a prototype for understanding protein kinase structure and function [1], and investigation of its sub‐cellular targeting has helped to establish general principles for the nanoscale organization of cell signalling. However, despite serving as a prototype, PKA possesses several unusual characteristics. Unlike other mammalian protein kinases, the catalytic and regulatory elements of PKA are expressed on separate polypeptides. Type I (RIα, RIβ) and type II (RIIα, RIIβ) PKA regulatory subunits are extremely abundant and in large excess of PKA catalytic (C) subunits in most tissues [2]. For example, in the cerebellum, RI subunits account for ~ 0.1% total protein and outnumber PKA catalytic subunits by ~ 17 to 1 [2]. All PKA regulatory subunits homo‐dimerize via unusual N‐terminal docking and dimerization (D/D) domains [3], with each regulatory subunit isoform assembling a tetrameric holoenzyme with a unique structure and sensitivity to cAMP. D/D domains also enable docking to A‐kinase anchoring proteins (AKAPs) for targeting to specific sub‐cellular regions [4]. Further quirks exhibited specifically by RI subunits are dimeric disulphide links in the D/D domain (Fig. 1, upper panel), and the ability to form condensates via liquid–liquid phase separation [5].

Protein kinase A regulates a wide variety of essential physiological processes, so it is no surprise that pathogenic mutations have been mapped to its subunits. These include activating mutations in C subunits that underlie Cushing's syndrome, such as Cα L205R [6]. Mutations in RIα that lead to nonsense‐mediated mRNA decay or increased sensitivity to cAMP are a common feature of Carney complex disorder [7]. In addition, the R335W mutation in RIβ has been linked to a neurodevelopmental disorder [8]. This mutation falls within the second cyclic nucleotide binding domain and probably reduces cAMP sensitivity [9]. In 2014, a pathogenic mutation was mapped to the D/D domain of RIβ: RIβ L50R substitution was found to coincide with a late‐onset neurodegenerative disorder presenting with dementia and/or parkinsonism [10], with RIβ L50R forming inclusions, particularly in the granular layer of the cerebellum [10]. A follow‐up study solidified a causal link between RIβ L50R mutation and this disease phenotype featuring aggregated RI subunits, leading to its naming as neuronal loss and parkinsonism driven by a PKA mutation (NLPD‐PKA) [11].

## The effects of L50R substitution in RIβ extend to altered catalytic subunit control

In this issue of *The FEBS Journal*, Benjamin‐Zukerman *et al*. [12] take our understanding of RIβ L50R pathogenicity a step further. They use a wide array of techniques to explain in detail how the substitution triggers not only RIβ aggregation but also an unexpected change in sensitivity of type Iβ PKA holoenzymes to cAMP with knock‐on effects on gene expression. The authors start by comparing the propensity for aggregation in four variants of RIβ that contain potentially pathogenic single nucleotide polymorphisms in the D/D domain. Reducing and nonreducing gels show that L50R is the only one of these substitutions to markedly shift RIβ from disulphide‐linked dimers to monomeric aggregates [12]. Inspection of the crystal structure of the wild‐type (WT) RIβ D/D domain shows that L50 lies within the second α‐helix, where it is buried within the heart of the D/D domain (Fig. 1, upper panel). Across the D/D domain superfamily, which includes many members beyond PKA regulatory subunits, only four amino acids are present at the equivalent position to RIβ L50: leucine, methionine, alanine and serine [3]. Longer, positively charged arginine is not compatible with the key role of position 50 in dimeric packing (Fig. 1, lower panel). Circular dichroism measurements show that the α‐helical content of the D/D approximately halves when the L50R substitution is present, indicating that the dimer interface and/or disulphide are important for maintaining secondary structure in the D/D.

The authors take advantage of patient‐derived fibroblasts to show that not only is the L50R variant more prone to forming aggregated monomers as expected, but that PKA C subunits are more easily mobilized to the nucleus in cells from L50/L50R patients. The difference in C subunit accumulation in the nucleus is most evident with 1 μm isoproterenol, a relatively naturalistic β‐adrenoreceptor agonist. Consistent with this finding, bioluminescence resonance energy transfer assays in transfected HEK293 cells show that there is a greater initial release of PKA C subunits from the L50R variant compared with WT RIβ. Furthermore, surface plasmon resonance experiments with purified PKA subunits reveal that the L50R mutation clearly increases catalytic subunit release at mid to low nanomolar concentrations of cAMP. A difference in propensity to release C subunits for nuclear import hints at scope for changes in gene expression in NLPD‐PKA, and the authors explore this notion by comparing transcriptomes in primary fibroblasts taken from a patient with NLPD‐PKA and a matched control. Although this analysis is limited to two individuals, the results are interesting. Approximately equal numbers of genes are up vs downregulated in the NLPD‐PKA samples, and downregulated genes include those encoding glutamate receptor subunits that are essential for glutamatergic synaptic transmission.

## Conclusion and future perspective

Why would disruption of the RIβ D/D interface alter C subunit releases? The answer lies in the complex inter‐subunit interactions that occur in intact PKA holoenzymes, including those assembled by RIβ [13]. Breaking these interactions alters cAMP sensitivity and the cooperativity of cAMP activation [1]. For example, mutations that disrupt homodimer interactions between RIα protomers have been implicated in Carney complex Disorder [14]. There is still much to be learned about the pathogenicity of NLPD‐PKA. For example, it is not clear exactly how RIβ L50R exerts its effects in the cerebellum—are transcriptional changes in this context important? Hopefully, the research can be leveraged to develop therapies to counteract the deleterious effects of RI aggregates and aberrant transcriptional regulation that appear to be key pathological features of NLPD‐PKA. Further instances of RI dysfunction in other neurodevelopmental disorders may also be uncovered in time [9]. Research on NLPD‐PKA adds to a series of recent fundamental discoveries concerning RI subunits, including the identification of RI‐selective anchoring proteins [15], skewed R>C expression ratios [2] and RI liquid droplets [5]. I expect quirky PKA to continue to surprise for some time yet.

## Conflict of interest

The author declares no conflict of interest.
